# Enhancing yogurt quality with a high-EPS-producing space mutant *Lactiplantibacillus plantarum* LP-Wang25: integrated assessment of yogurt rheological behavior, flavoromics, and functional activities

**DOI:** 10.3389/fmicb.2026.1943949

**Published:** 2026-09-24

**Authors:** Lingxiang Wen, Youming Tan, Sizhe Qiu, Zhennai Yang, Hong Zeng, Yanbo Wang

**Affiliations:** 1 Key Laboratory of Geriatric Nutrition and Health, Beijing Technology and Business University, Ministry of Education, Beijing, China; 2 School of Food and Health, Beijing Technology and Business University, Beijing, China; 3 State Key Laboratory of Bioreactor Engineering, East China University of Science and Technology, Shanghai, China

**Keywords:** adjunct culture, antioxidant/anti-inflammatory activity, exopolysaccharide (EPS), *Lactiplantibacillus plantarum*, whole genome sequencing, yogurt flavor

## Abstract

**Introduction:**

Exopolysaccharides (EPS) can serve as thickeners, emulsifiers, and gelling agents, and have been widely applied in the food industry. *In situ* production of EPS by lactic acid bacteria has emerged as a promising alternative to the addition of exogenous EPS, aligning with the growing demand for clean-label foods.

**Methods:**

The application potential of a space mutant of *Lactiplantibacillus plantarum*, designated LP-Wang25, as the adjunct starter culture was comprehensively examined via EPS quantification, carbon source utilization assays, whole-genome sequencing, antioxidant and anti-inflammatory activity assays, yogurt fermentation trials, sensory evaluation, and volatile flavor analysis.

**Results:**

LP-Wang25 exhibited a shortened lag phase, 39.7% higher EPS production (161.95 mg/L), and enhanced antioxidant and anti-inflammatory activities compared with the parental strain. Whole-genome sequencing showed that EPS biosynthesis mainly proceeds through a conserved Wzx/Wzy-dependent pathway, indicating the increased EPS production is likely associated with transcriptional regulation and metabolic flux redistribution. When used as an adjunct starter, LP-Wang25 significantly improved yogurt quality by increasing EPS content and viscosity, reducing whey separation, enhancing milky aroma, acetoin concentration and sensory acceptability.

**Conclusion:**

LP-Wang25 represents a promising adjunct culture for producing clean-label yogurt with improved rheological and sensory attributes as well as enhanced antioxidant and anti-inflammatory activities. This study bridges space-induced microbial breeding with tangible dairy industry applications, paving the way for next-generation functional fermented products.

## 1 Introduction

Yogurt, a popular fermented dairy product, is distinguished by its unique sensory characteristics and nutritional density, including high-level proteins, vitamins, minerals and probiotics (Ahmad et al., 2022; Wang et al., 2025b), making it an important component of modern healthy diet. Among various sensory attributes, texture is one of the key factors influencing yogurt quality and consumer acceptance (Le Ba et al., 2025). High-quality yogurt is typically characterized by a smooth and viscous gel-like structure (Zhao et al., 2023), with minimal whey separation during storage. Rheological and textural parameters such as hardness, stickiness, cohesiveness, viscosity, gel strength, and elasticity are critical indicators for quantitative evaluation of yogurt quality (Le Ba et al., 2025). The formation of an ideal yogurt texture is a complex physicochemical process (Beldring et al., 2026). While the texture is influenced by the raw milk matrix composition, the functional properties of the starter cultures, particularly their contribution to the construction of the gel network during fermentation, play a decisive and critical role (Le Ba et al., 2025; Beldring et al., 2026).

Exopolysaccharides (EPSs), being a natural bioactive biopolymer, have been extensively employed in the food industry as thickeners, emulsifiers and gelling agents (Kaur and Dey, 2023; Netrusov et al., 2023). While commonly utilized EPSs such as xanthan, dextran and pectin are effective (Kaur and Dey, 2023; Netrusov et al., 2023), they are usually *ex situ* produced (i.e., isolation and purification of EPS) and labeled as food additives. Driven by the rising demand for clean-label products, the *in situ* production of EPS by lactic acid bacteria (LAB) has emerged as a promising alternative (Korcz and Varga, 2021). Mechanistically, LAB-derived EPS improves yogurt texture through complex physicochemical interactions. Free EPS, secreted into the fermentation matrix, is rich in hydroxyl and carboxyl groups that facilitate water-holding capacity and reduce whey separation; interacts with casein micelles via hydrogen bonding and electrostatic interactions, thereby stabilizing the protein network (Liang et al., 2024; Zang et al., 2025). Capsular EPS, which is bound to cell surface, interacts with proteins through direct contact forming compact and localized EPS-protein complexes, there-by strengthen gel structure and shear resistance (Zang et al., 2025). Moreover, through steric hindrance, EPS can prevent excessive protein aggregation and phase separation, enhancing the gel’s resistance to shear forces and syneresis. A study indicated that compared to exogenous additives, the consistency index was higher with yogurt containing *in situ* produced EPS (Doleyres et al., 2005). In addition, LAB-derived EPSs exhibit antioxidant activity by scavenging free radicals and chelating metal ions, and they also exert anti-inflammatory effects by modulating cytokine expression through TLR signaling pathways. These bioactivities offer potential health benefits beyond the texture-improving role of EPS in yogurt (Xu et al., 2024).

However, the industrial application of *in situ*-produced EPS is often hindered by the low and unstable yield of wild-type LAB strains (Korcz and Varga, 2021). Screening for high-yield isolates from natural sources is labor-intensive, while yield enhancement via genetic engineering or synthetic biology faces strict food safety regulations (Ottenheim et al., 2018). Consequently, microbial breeding through mutagenesis remains an effective strategy. Space mutagenesis has emerged as a novel breeding approach (Tan et al., 2026a). For example, space-derived mutants of LAB have exhibited enhanced growth, probiotic functions (Yang et al., 2023), anti-inflammatory activity (Sun et al., 2018), and pectin-degrading enzyme activity (Song et al., 2023). Notably, space mutagenesis has also enabled EPS production in mutant strains derived from a non-producing parental strain (Liu et al., 2023).

*Lactiplantibacillus plantarum* is a versatile species widely used in dairy, meat and vegetable fermentation for its functional properties and its bioactive EPS (Jiang and Yang, 2018; Xiong et al., 2023). Our previous work reported a high-EPS-producing strain, *L. plantarum* HXM2, isolated from traditional Chinese sauerkraut, possessed a maximum yield of 556.43 mg/L under optimized fermentation conditions (Hao et al., 2025). In this study, we evaluated the application potential of a mutant strain of HXM2, i.e., named as LP-Wang25 obtained via space mutagenesis, being an adjunct culture to enhance yogurt quality. First, the physiological differences between the mutant and parental strains were characterized by comparing their growth kinetics and EPS production. Subsequently, whole genome sequencing was used to identify genomic variations associated with EPS biosynthesis. The bioactivity of LP-Wang25-produced EPS was evaluated through antioxidant activity and anti-inflammatory activity assays. Furthermore, the mutant strain was applied for yogurt fermentation, and the *in situ* EPS yield, rheological properties, flavoromics and sensory attributes were examined via rheometer, gas chromatography-ion mobility spectrometry (GC-IMS) and quantitative descriptive analysis (QDA).

## 2 Materials and methods

### 2.1 Isolation of single cell mutant colony

The parental strain HMX2 is currently preserved at the China General Microbiological Culture Collection Center (CGMCC) and Beijing Technology and Business University. For preflight preparation, HMX2 was streaked onto MRS agar containing 1.5% agar. Upon completion of the spaceflight, the samples were serially diluted and plated to obtain isolated bacterial colonies for subsequent analysis.

### 2.2 Growth characteristics of strains

The space mutant strain LP-Wang25 and the wild-type strain HMX2 were activated by incubation in MRS medium at 37 °C for 24 h, followed by three successive subcultures at 37 °C for 12 h each. The growth curves of the activated strains were continuously monitored for 24 h at 37 °C using an automated growth curve analyzer (at OD 600 nm; MGC200, China).

### 2.3 Biolog EcoPlate analysis

The carbon source utilization profiles of the mutant strain LP-Wang25 and the parental strain HMX2 were evaluated using a Biolog EcoPlate™ (Biolog Inc., Hayward, CA, USA). The EcoPlate™ contains different carbon sources with water included as a negative control. Each well contains tetrazolium dyes that act as a redox indicator by accepting electrons generated during microbial respiration, resulting in a progressive color development that reflects microbial metabolic activity. According to the manufacturer’s instruction, 1 mL of activated culture from Section 2.2 was diluted to an optical density of 0.2 at 600 nm. Prior to inoculation, the EcoPlate™ was equilibrated to 25 °C. Subsequently, 150 μL of the diluted cell suspension was inoculated into each well. The plates were incubated at 28 °C for 240 h. Absorbance was read at 590 nm on the Multiskan MK3 microplate reader (Thermo Fisher Scientific, Waltham, MA, USA).

### 2.4 Determination of EPS content by phenol-sulfuric acid methods

EPS produced by both strains in MRS broth and yogurt samples were determinate by phenol-sulfuric acid methods.

Activated strains were inoculated into fresh MRS broth at 2% (v/v) and cultured at 37 °C for 24 h. The cultures were then heated at 100 °C for 20 min and then centrifuged at 7,000 rpm for 20 min at 4 °C. Trichloroacetic acid was added to the supernatant to a final concentration of 40 g/L, followed by storage at 4 °C for 12 h to precipitate proteins. After centrifugation under the same conditions, the supernatant was mixed with 95% ethanol (3:1 v/v) and stored overnight at 4 °C to precipitate EPS. The precipitate was collected by centrifugation with same conditions, redissolved in deionized water and dialyzed (14 kDa cur off, 4 °C, 48 h, water changed every 8 h). The dialysate was concentrated and vacuum freeze-dried. The lyophilized EPS was redissolved in deionized water for further analysis.

EPS extraction from yogurt samples followed the same procedure, with an additional defatting step. After protein precipitation and centrifugation, the supernatant was mixed with a chloroform–n-butanol solution (4:1, v/v), vigorously shaken for 5 min, and centrifuged at 8,000 rpm for 15 min at 4 °C. The supernatant was collected and subjected to subsequent EPS precipitation and purification steps as described above.

The EPS concentration in the redissolved suspension after dialysis was quantified using the phenol-sulphuric method of Charchoghlyan et al. (2017), and is expressed as glucose equivalent with glucose as the standard.

### 2.5 Whole genome sequencing

Whole genome sequencing was conducted to identify potential mutations in the mu-tant strains. The parental strain and the mutant strain were cultured in MRS broth at 37 °C for 14 h. Cells were harvested by centrifugation, washed twice with sterile saline, and collected (≥0.1 g wet weight). Total genomic DNA extraction and subsequent whole genome sequencing were performed by Shanghai Majorbio Bio-Pharm Technology Co., Ltd. Whole genome sequencing of strains was performed using the Illumina NovaSeq 6,000 platform with 150 bp pair-end library construction. Raw sequencing reads were processed through quality control and adapter trimming by fastp (v 0.20.0). Cleaned reads were aligned to the reference genome using BWA-MEM. single nucleotide polymorphisms (SPNs) and insertions and deletions (InDels) were identified with snippy (v 4.6.0), followed by annotation of variant sites using SnpEff, which included mutation type, genomic location, and potential impact on protein coding.

The genome of strain HXM2 was selected as the reference genome. The predicted genes were functionally annotated in COG databases (Galperin et al., 2024), KEGG database (Kanehisa et al., 2024) and CAZyme database (Drula et al., 2021).

### 2.6 Assessment of the antioxidant and anti-inflammatory properties of EPS

#### 2.6.1 2,2-Diphenyl-1-picrylhydrazyl (DPPH) and hydroxyl radical scavenging assays

The DPPH radical scavenging activity of the EPS was evaluated following the method described by Tarannum et al. (2024). Briefly, 1.0 mL of the ethanolic EPS solution was mixed with an equal volume of 0.1 mM ethanolic DPPH solution, and the mixture was vortexed thoroughly. After incubation at room temperature in the dark for 30 min, the absorbance was measured at 517 nm using a microplate reader (Flash SuperMax 3,200, Shanghai, China), with a reagent blank serving as the reference. Ascorbic acid was used as positive control. The DPPH radical scavenging activity was calculated according to the following equation:


$$\begin{array}{l} \text{DPPH radical scavenging activity}\;(\%)=\\ {}[1-(A_{\text{sample}}-A_{\text{blank}})/A_{\text{control}}]\times 100 \end{array}$$

where *A*_sample_, *A*_blank_, and *A*_control_ denote the absorbances of the sample, the blank, and the control, respectively.

The hydroxyl radical scavenging activity was assessed following a modified version of the salicylic acid method (Nemati and Mozafarpour, 2024). 1.0 mL of crude EPS solution, 1.0 mL of 5 mM salicylic acid-ethanol solution, and 1.0 mL of 5 mM FeSO₄ solution were mixed and then added 1.0 mL of 3 mM H₂O₂ and 6.0 mL of distilled water. After mixing thorough, the mixture was incubated at 37 °C for 15 min, and the absorbance was measured at 510 nm (Flash SuperMax 3,200, Shanghai, China). Ascorbic acid was used as positive control. The blank was prepared by substituting the EPS solution with distilled water. The hydroxyl radical scavenging activity was calculated using the following formula:


$$\begin{array}{l} \text{Hydroxyl radical scavenging activity}\;(\%)=\\ {}[1-(A_{\text{sample}}-A_{\text{blank}})/A_{\text{control}}]\times 100 \end{array}$$

where *A*_sample_, *A*_blank_, and *A*_control_ represent the absorbances of the sample, the blank, and the control, respectively.

#### 2.6.2 Determination of anti-inflammatory activity

Anti-inflammatory activity was determined using the method described by Tarannum et al. (2024). A mixture consisting of 2.0 mL of crude EPS solution, 0.2 mL of egg albumin, and 2.8 mL of 10 mM PBS was incubated at 37 °C for 15 min, followed by incubation in a 70 °C water bath for 5 min. After cooling to room temperature, absorbance was measured at 600 nm using a microplate reader (Flash SuperMax 3,200, Shanghai, China). Using Aspirin as control. The anti-inflammatory activity was calculated according to the following equation:


$$\begin{array}{l} \text{Anti}-\text{inflammatory activity}\;(\%)=\\ {}[(A_{\text{control}}-A_{\text{sample}})/A_{\text{control}}]\times 100 \end{array}$$

where *A*_sample_, and *A*_control_ represent the absorbances of the sample and the control, respectively.

### 2.7 Yogurt fermentation

After activation, strain LP Wang25 was co-inoculated with *Lactococcus lactis* BL19 (*L. lactis* BL19) (Guo et al., 2026) or with the commercial starter culture Yo-Mix® Prime 5.0 (Chr. Hansen, Hørsholm, Denmark) into whole fat milk based medium. For comparison, control fermentations were performed with *L. lactis* BL19 (Guo et al., 2026) alone and with Prime 5.0 alone under identical conditions. The medium was prepared by dissolving whole fat milk powder (Fonterra Ltd., Auckland, New Zealand; 26.2% fat, 40.3% lactose, and 24.5% protein) at a concentration of 11.5% (w/w) in distilled water, supplemented with 5.0% sucrose (Taigu Co., Ltd., Guangdong, China). The mixture was pasteurized at 95 °C for 5 min and then cooled to room temperature. Fermentation was carried out at 37 °C until the pH reached 4.6. All yogurt samples were stored at 4 °C.

### 2.8 Rheological analysis of yogurt

The rheological properties of the yogurt samples were analyzed using a rotational rheometer (HAAKE, Thermo Fisher Scientific, MA, USA). The shear rate was increased from 25 to 1,000 s^−1^.

### 2.9 Sensory evaluation of yogurt samples

A sensory descriptive analysis was conducted by eight trained panelists (4 females and 4 males, aged 20–30 years old) (Huang et al., 2026). The panel comprised non-smokers with no history of rhinitis and a minimum of 3 years of experience in dairy product sensory evaluation. All participants were fully informed of the study content and provided written informed consent. The sensory evaluation protocol was approved by the Scientific Research Ethics Committee of Beijing Technology and Business University (Approval No. 2025-239).

The assessment was carried out in individual sensory booths under controlled lighting and ambient conditions. Approximately 50 g of each yogurt sample was presented in white transparent plastic cups labeled with three-digit random codes. Low-mineral water and unsalted crackers were provided for palate cleansing between samples, and coffee beans were available to neutralize olfactory fatigue.

Panelists were instructed to evaluate each sample according to the provided sensory score sheet, which included ten attributes across three categories: appearance (color, gloss, whey separation), aroma/flavor (dairy aroma intensity, off-flavor, sweet–sour balance), and texture/mouthfeel (thickness, smoothness, adhesiveness, fluidity). Each attribute was rated on a 5-point intensity scale, with descriptive anchors provided for clarity. The evaluation was performed in a randomized order, and panelists were required to rinse thoroughly with water and wait at least 60 s before proceeding to the next sample.

### 2.10 Analysis of volatile flavor compounds in yogurt

The analysis was performed using a gas chromatography-ion mobility spectrometry (GC-IMS) instrument (Flavor Spec, Shandong Haineng Scientific Instrument Co., Ltd., China) with an MXT-5 capillary column (15 m × 0.53 mm i.d. × 1 μm). 1.8 mL of distilled water and 0.2 g of sample was added to a 20 mL headspace vial. Sa incubated at 60 °C for 10 min. The carrier gas flow rate was set as follows: the initial flow rate was 2 mL/min for 2 min, then increased to 10 mL/min at 10 min, further increased to 100 mL/min at 20 min, and finally increased to 150 mL/min at 30 min. The drift gas flow was maintained at 75 mL/min. n-ketones (C4-C9) were used to calculate the retention index (RI). The qualification of flavor compounds was achieved by comparing the RI data with the GC-IMS library and the NIST database.

### 2.11 Statistical analysis

All statistical analyses and data visualization were performed using SPSS (v24.0, IBM, Armonk, NY, USA) and Python (v. 3.12.3) in Jupyter Notebook environment. Multivariate analysis (PCA and OPLS-DA) was conducted using SIMCA (v14.1, Sartorius Stedim Bio-tech, Umeå, Sweden). All experiments were conducted in triplicate and data are presented as mean ± standard deviation (SD). Differences between groups were assessed using appropriate statistical tests. Statistical significance was defined as: *p < 0.05, **p < 0.01, and ***p < 0.001.

## 3 Results and discussion

### 3.1 Growth and carbon metabolic capability of the mutant strain LP-Wang25

The mutant strain LP-Wang25 exhibited a growth profile distinctly different from that of the parental strain HXM2 (Figure 1A). In MRS broth, the lag phase of LP-Wang25 was significantly shortened, which was consistent with previous reports (Horneck et al., 2010; Tan et al., 2026a). Specifically, the mutant entered the exponential phase after approximately 1 h and reached the stationary phase by 6 h, with no signs of the decline phase observed within the 24-h period. In contrast, the parental strain HXM2 displayed a prolonged lag phase lasting 15 h and failed to reach the stationary phase within the 24-h period. Given that HXM2 is characterized by high EPS production (Hao et al., 2025; Hu et al., 2025), we investigated the variations in EPS production between the mutant and the parental strains. As shown in Figure 1B, after 24 h of growth in MRS broth, the EPS production significantly exceeded that of the parental strain (*p* < 0.001), reaching 161.95 mg/L. This represented a 39.7% increase over 115.90 mg/L yield of parental strain.

**Figure 1:**
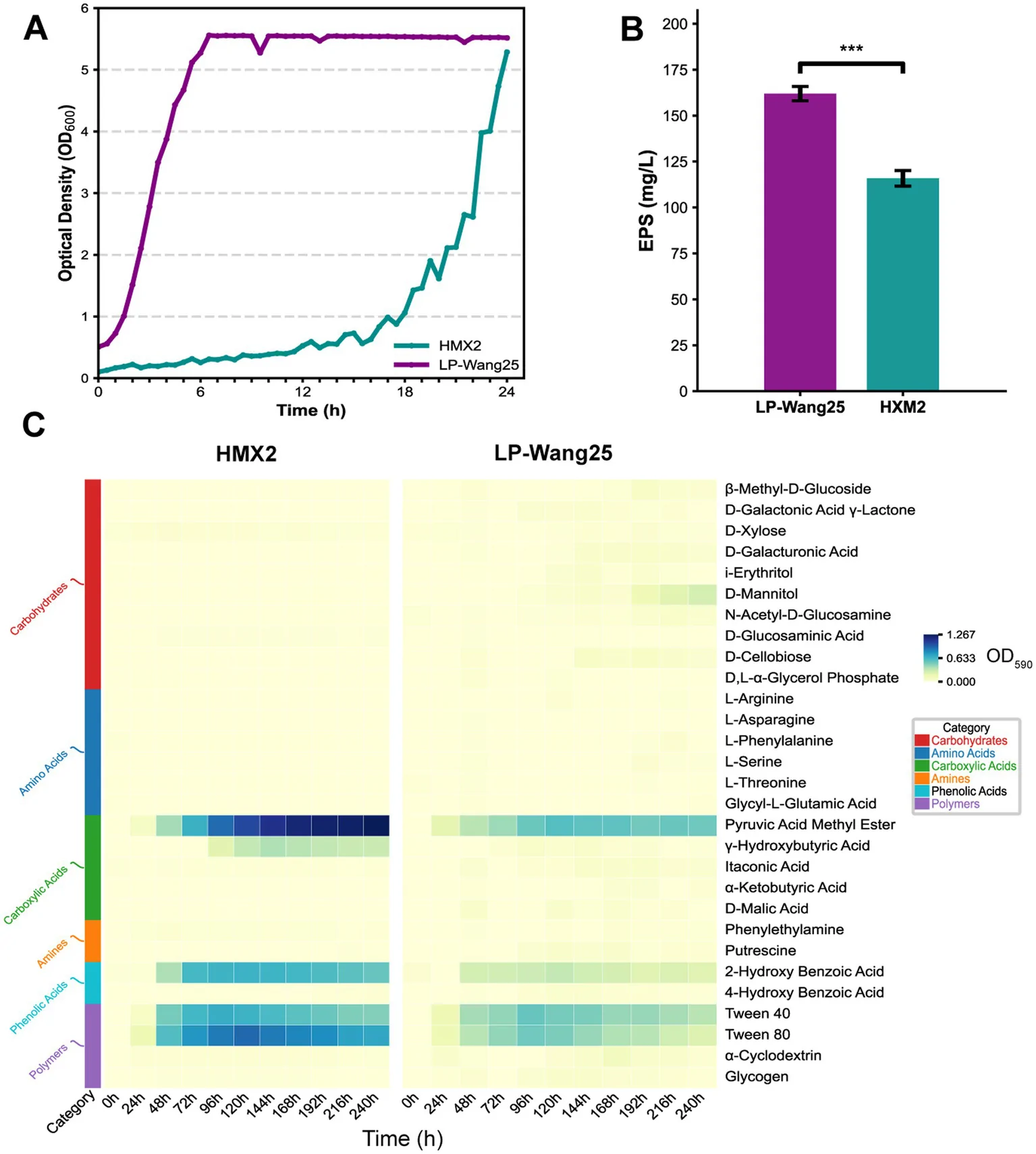
Comparison of growth and carbon metabolic capability between the mutant strain LP-Wang25 and the parental strain HMX2: **(A)** growth curves in MRS medium, **(B)** EPS production in MRS medium, and **(C)** carbon source metabolic capability.

Carbon metabolic capability of the mutant strain was evaluated using Biolog EcoPlates. Generally, the mutant strain exhibited a lower overall metabolic activity and a distinctive patten compared to the parental strain (Figure 1C). Similarly, a previous study found a different metabolism of carbon sources between space mutant strains and parental strains (Sun et al., 2024). The parental HXM2 utilized γ-hydroxybutyric acid, whereas the mutant strain could not. The mutant strain displayed a potential ability to metabolize D-mannitol (OD_590_ = 0.258 on day 10) while the parental strain could not. Among the carbon sources tested, strain HXM2 exhibited the highest growth intensity on pyruvic acid methyl ester, with the maximum OD observed on day 10 (OD_590_ = 1.267). The mutant strain showed a much lower growth intensity on the same carbon source, reaching its maximum OD on day 5 (OD_590_ = 0.580). As for polymers, both strains effectively utilized Tween 40 and Tween 80, showing a growth kinetic pattern of initial increase followed by a decline, a maximum OD at 96 h. Since Tweens are polysorbates consisting of different fatty acid (palmitic acid in Tween 40 and oleic acid in Tween 80), their utilization indicates high lipase activity (Dzionek et al., 2025), consistent with previous reports describing *L. plantarum* as a good source of lipase (Lopes et al., 2002; Aiello et al., 2023). The growth of the mutant on Tween 80 (OD_590_ = 0.511, at 96 h) was lower than that of the parent (OD_590_ = 0.919, at 96 h), suggesting a reduced efficiency in utilizing long-chain fatty acids. Neither strain utilized glycogen or α-cyclodextrin, likely due to the absence or insufficient expression of extracellular α-glucan degrading enzymes, such as α-amylase or cyclodextrinase.

### 3.2 Genomic analysis of the mutant strain LP-Wang25

Whole genome sequencing analysis revealed that the genome size of both strains is approximately 3.3 Mbp, containing ~3,100 protein-coding genes (Supplementary Table S1). To evaluate the genomic variations induced by space mutagenesis, the sequencing reads of the mutant strain LP-Wang25 were aligned against the parental strain HXM2, which served as the reference genome. The alignment results indicated an average depth of 305.76× and a genome coverage of 96.60%.

A total of 14,723 variants were identified across the genome. The detailed mutation types and their predicted effects are summarized in Table 1. SNPs were identified as the predominant variation type in the LP-Wang25 genome which comprised transitions and transversions. This observation is consistent with previous findings that spaceflight mutagenesis primarily induces point mutations (Deyong et al., 2025). Variation effect analysis revealed that the SNPs were mostly located in non-coding regions, such as upstream and downstream of genes, suggesting potential impacts on gene expression via transcriptional regulation rather than direct alteration of protein sequence and structures. Within the coding sequences, most SNPs were synonymous mutations (Figure 2A), while high-impact variations, such as missense and nonsense mutations, were less frequent. InDels involve the insertion and deletion of one or more nucleotides and have a higher potential to disrupt biological functions by altering open reading frames. Similarly, the InDels in the LP-Wang25 genome were largely located in non-coding regions (Figure 2B). Only a small fraction of InDels (36 variants, accounting for 1.2%) were located in the coding sequences and predicted to be frameshift mutations. This suggests that despite the high mutation load, the structural integrity of most functional proteins remained largely preserved.

**Table 1:** Variant types and effects in LP-Wang25.

Variant type	Number
SNP	13,012 (88.4%)
MNP	1,354 (9.2%)
InDel	276 (1.9%)
MIXED	81 (0.5%)

Variant effects type	Number
Inframe deletion	20 (0.014%)
Downstream variant	66,440 (45.14)
Initiator codon variant	5 (0.003%)
Intron variant	1 (0.001%)
Splice region variant	44 (0.03%)
Stop gained	18 (0.012%)
Stop retained variant	23 (0.016%)
Upstream variant	65,945 (44.80%)
Inframe insertion	18 (0.012%)
Frameshift variant	39 (0.026%)
Intergenic region	3,098 (2.105%)
Missense variant	3,430 (2.33%)
Start lost	2 (0.001%)
Stop lost	20 (0.014%)
Synonymous variant	8,084 (5.492)

SNP, single nucleotide polymorphism; MNP, multiple nucleotide polymorphism; InDel, insertion and deletion; MIXED, multiple nucleotides and an InDel.

**Figure 2:**
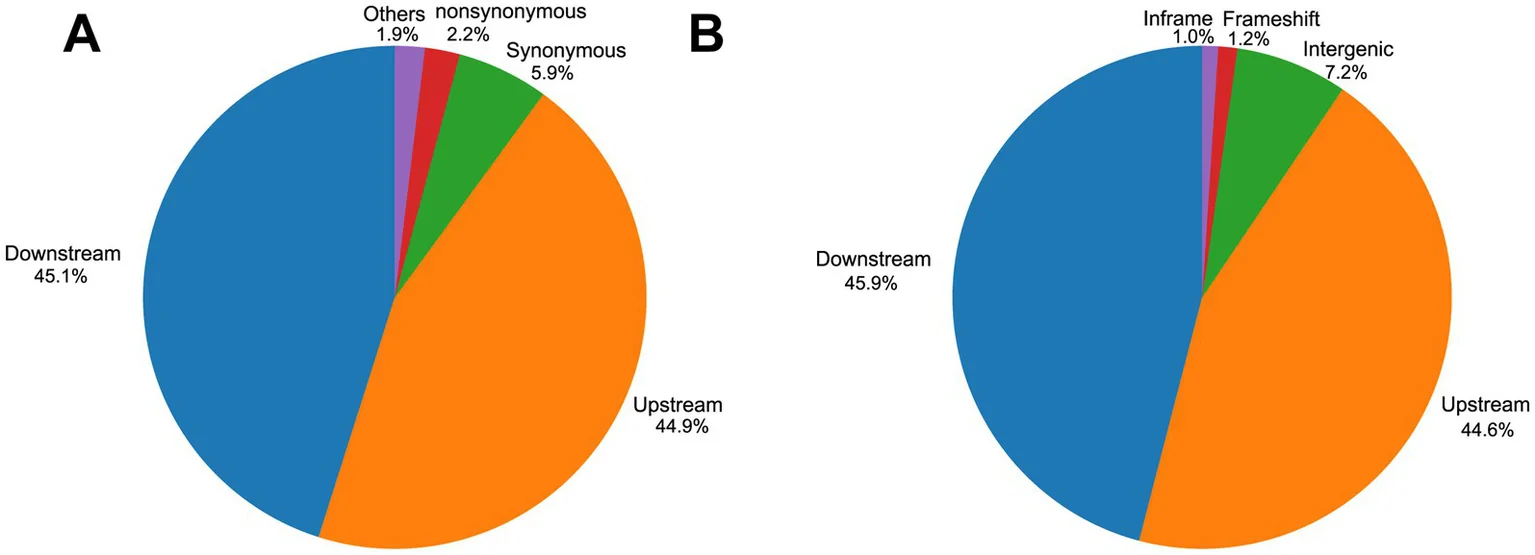
Genome variations effects of genome of LP-Wang25. **(A)** SNP-induced mutation results. Nonsynonymous includes missense mutations and nonsense mutations. **(B)** InDel-induced mutation results.

EPS biosynthesis in LAB is primarily via four pathways, namely the extracellular synthesis pathway, the synthase-dependent pathway, the ATP-binding cassette (ABC) transporter-dependent pathway, and the Wzx/Wzy-dependent pathway (Bibi et al., 2021). Based on functional screening of genes associated with these mechanisms, genes involved in EPS biosynthesis were listed in Table 2, Supplementary Table S2. The extracellular synthesis pathway relies on extracellular sucrases to synthesize homopolysaccharides (HoPS), including glucans and fructans (Wu et al., 2023). These enzymes are glycoside hydrolases (GH) and usually belong to GH68 and GH70 families (Lynch et al., 2018). A total of 54 genes were annotated as GHs (Supplementary Table S3); however, none of these genes belonged to the GH68 or GH70 families. This indicates that both strains lack the extracellular synthesis pathway. The other three pathways involve intracellular synthesis followed by export via distinct mechanisms. In the synthase-dependent pathway, a specialized synthase (a subunit of envelope-spanning multiprotein complex in some cases) simultaneously catalyzes polymer chain elongation and translocation across the membrane. A classic example is hyaluronan synthase, which polymerizes glucuronic acid and GlcNAc into hyaluronan (Schmid et al., 2015). However, no homologous genes associated with this pathway were identified in the genome. The third mechanism is the ABC transporter-dependent pathway, which is primarily responsible for the biosynthesis of capsular polysaccharides (Schmid et al., 2015). In this pathway, the polysaccharide chain is continuously assembled and extended by glycosyltransferases (GTs). Subsequently, the polymer is exported via a tripartite efflux pump complex, typically composed of an ABC transporter in the cytoplasmic membrane, a polysaccharide co-polymerase (PCP), and an outer membrane polysaccharide export (OPX) protein, in gram-negative strains (Schmid et al., 2015). In gram-positive strain, the export of polysaccharide can directly via ABC-transporter. Many genes were annotated as ABC transporter ATP-binding protein and ABC transporter permease (Supplementary Table S2), some of which were likely involved in this pathway.

**Table 2:** Genes identified in the EPS gene cluster of LP-Wang25.

Gene ID	Gene	Encoding protein	Length (bp)	Variant type^b^	Variant effect
Gene0778	*cpsG*	glycosyltransferase, family 1 (GT1)	1,175	SNP, MNP	Synonymous variant, missense variant
Gene0884	*epsH*	glycosyltransferase, family 2	732	–^c^	–
Gene2139	*cps3H*^a^	UniProt: polysaccharide biosynthesis protein (F9UN19)	1,109	SNP, MNP	Synonymous variant, missense variant
Gene2140	*cps3F*^a^	UniProt: polysaccharide polymerase (F9UN17)	1,196	SNP	Synonymous variant, missense variant, initiator codon variant
Gene2141	*cps3E*^a^	UniProt: polysaccharide biosynthesis protein (F9UN16)	623	SNP, MNP	Synonymous variant, missense variant, start lost, intron variant
Gene2142	*cps3D*^a^	UniProt: polysaccharide biosynthesis protein (F9UN15)	1,088	SNP, MIXED	Synonymous variant, missense variant, stop lost, inframe deletion
Gene2143	*glf*	UDP-galactopyranose mutase	1,133	SNP	Synonymous variant, missense variant
Gene2144	*glf1*	glycosyltransferase, family 2	932	–^c^	–
Gene2145	*glf1*	glycosyltransferase, family 2	911	–^c^	–
Gene2164	–	oligosaccharide flippase family protein	1,382	–^c^	–
Gene2165	*epsI*	UniProt: polysaccharide pyruvyl transferase family protein (A0ABT0M8H2)	1,073	–^c^	–
Gene2166	*epsE*	UniProt: glycosyltransferase family 2	959	–^c^	–
Gene2167	*cps4H*^a^	UniProt: polysaccharide polymerase (F9UQ48)	1,274	–^c^	–
Gene2168	*wekW*	glycosyltransferase	1,031	–^c^	–
Gene2169	*epsD*	glycosyltransferase family 4 protein	1,094	–^c^	–
Gene2170	*wecP*	UDP-GalNAc: undecaprenyl-phosphate GalNAc-1-phosphate transferase	677	–^c^	–
Gene2171	*galE*	UDP-glucose 4-epimerase	947	–^c^	
Gene2172	*cps2C*^a^	UniProt: Tyrosine-protein phosphatase (F9UMZ7)	773	SNP, MNP	Synonymous variant, missense variant
Gene2173	*epsB*	tyrosine-protein kinase	728	SNP, MNP	Synonymous variant, missense variant
Gene2174	*epsA*	Wzz/FepE/Etk N-terminal domain-containing protein	770	MNP	Synonymous variant
Gene2176	*rfbB*	dTDP-glucose 4,6-dehydratase	1,016	–^c^	–
Gene2179	*wzx*	flippase	1,415	–^c^	–
Gene2180	*glf1*	glycosyltransferase family 2 protein	1,112	None	None
Gene2182	*waaB*	lipopolysaccharide 1,6-galactosyltransferase	1,064	None	None
Gene2483	*cps4A*	UniProt: capsular polysaccharide biosynthesis protein (F9UQ55)	758	–^c^	–
Gene2484	*epsB*	tyrosine-protein kinase	707	SNP	Stop gain, missense variant
Gene2485	*cps4C*^a^	UniProt: tyrosine-protein kinase (F9UQ53)	821	SNP	Missense variant
Gene2486	*galE*	UDP-glucose 4-epimerase	941	SNP	Synonymous variant
Gene2487	*wecP*	UDP-GalNAc: undecaprenyl-phosphate GalNAc-1-phosphate transferase	665	SNP	Synonymous variant, missense variant
Gene2488	*epsD*	glycosyltransferase family 4 protein	1,091	SNP	Synonymous variant, missense variant
Gene2489	*cps4G*^a^	UniProt: glycosyltransferase, family 1 (GT1) (F9UQ49)	1,028	SNP, MNP	Synonymous variant, missense variant
Gene2490	*cps4H* *^a^*	UniProt: polysaccharide polymerase (F9UQ48)	1,274	SNP, MNP	Synonymous variant, missense variant
Gene2491	*epsE*	glycosyltransferase	968	SNP	Synonymous variant, missense variant
Gene2492	*cps4J*^a^	UniProt: polysaccharide repeat unit transporter (flippase)	1,451	SNP	Synonymous variant, missense variant

a Gene name and protein name obtained by a best BLAST hit in the UniProtKB and Swiss-Prot databases. The code in parentheses refers to the UniProt entry.

b Only variations in the gene sequence are listed; SNP or other variations in other locations, such as upstream and downstream, are not listed.

c No variation in the gene sequence but has variations in other location.

The final pathway is the Wzx/Wzy-dependent pathway, a complex process typically responsible for the biosynthesis of heteropolysaccharides (HePS) through five sequential steps. The first step involves sugar uptake and phosphorylation, where carbon sources like glucose, lactose, and galactose are transported intracellularly via permeases or the phosphotransferase system (PTS). A large number of genes involved in this step were identified (Supplementary Table S2), such as gene0026 and gene0881 (transport lactose), gene0936 and gene0999 (transport glucose). The second step is the formation of sugar nucleotides, where phosphorylated sugars are converted into activated precursors (e.g., UDP-Glucose, UDP-Galactose and dTDP-Rhamnose) by various isomerases and pyrophosphorylases (Laws et al., 2001; Wu et al., 2023). Genes involved in this step were identified (Supplementary Table S2), such as gene2495 (1-phosphofructokinase), gene2953 (6-phosphofructokinase) and gene3100 (rhamnose isomerase) etc. The third step is the assembly of repeating units, in which GTs sequentially assemble monosaccharides onto an undecaprenol diphosphate anchor located on the inner membrane (Zhou et al., 2019). The GTs were also largely identified (Supplementary Table S3). The fourth step is translocation, where the lipid-linked repeating units are flipped from the intracellular to the extracellular of the membrane by a flippase (Wzx) (Islam and Lam, 2013; Zhou et al., 2019). The fifth step is polymerization, where the repeating units are polymerized into long chains by a polymerase (Wzy) at the extracellular interface. Two EPS gene clusters were found in the reference genome (Table 2), which primarily encode enzymes involved in the latter three steps, as enzymes in the first two steps are typically not encoded within the EPS cluster. The second cluster is shorter, mainly encoding GTs (gene2488, gene2489, gene2491), a flippase (gene2492), and a polymerase (gene2490). The first cluster is larger, containing multiple GTs (gene2166, gene2168, gene2169, gene2180), flippases (gene2164, gene2179), and a polymerase (gene2167). The *epsA* (gene2174) within this cluster is predicted to regulate chain length and export (Lu et al., 2024). Additionally, this cluster includes genes involved in the conversion of monosaccharides to sugar nucleotides (e.g., gene2170, gene2171, gene2176, gene2182). The *epsB* encodes a protein likely involved in ATP-dependent regulatory steps during EPS synthesis, such as polymerization and transmembrane transport (Huang and Schell, 1995), with similar functions predicted for gene2172, gene2484, and gene2485.

Genomic variation analysis revealed that most genetic variations were located in the upstream or downstream regions of EPS-related genes, with only a small fraction occurring within the coding sequences (Supplementary Table S4). The variations within the coding regions were primarily nucleotide substitutions (SNPs and MNPs), resulting mainly in synonymous and missense mutations. Synonymous mutations do not alter the amino acid sequence and thus preserve protein function. The observed missense mutations, although causing amino acid substitutions, were predicted to have limited impact on protein function as the affected sites are likely outside the core catalytic domains. Among the relevant genes, only gene2141 and gene2412 underwent high-impact mutations, specifically a start codon loss and a frameshift mutation, respectively, which could potentially impair the synthesis and function of the corresponding proteins. However, no reduction in EPS synthesis capacity was observed, which is likely due to the presence of functional redundancy provided by paralogous gene copies in the genome (gene2139 and gene2483).

Overall, the above results suggested that mutagenesis did not introduce significant destructive variations in the coding regions of core EPS biosynthetic enzymes, indicating the high conservation of the EPS gene clusters (Zeidan et al., 2017). Therefore, the enhanced EPS yield in the mutant strain is likely attributed to mutations in non-coding regions or regulatory genes, which may upregulate the expression levels of EPS-related genes. Furthermore, variations in genes related to sugar transformation and transport (Supplementary Table S2) may enhance the capacity for glucose or lactose uptake of the mutant strain, thereby increasing the supply of sugar nucleotide precursors for EPS synthesis. Concurrently, mutations in regulatory genes may lead to an increased carbon flux allocated to EPS biosynthesis pathway over the glycolytic pathway used for cell growth.

### 3.3 Characterization of antioxidant and anti-inflammatory activities of EPS

The DPPH radical scavenging activities of HMX2-EPS and LP-Wang25-EPS were determined to be 93.75 ± 0.06% and 89.65 ± 0.10%, respectively. And their hydroxyl radical scavenging activities were 91.04 ± 0.05% and 85.47 ± 0.04%, respectively. These results indicated that EPS from both strains exhibited good *in vitro* antioxidant activity, with LP-Wang25-EPS demonstrating better antioxidant performance.

The anti-inflammatory activity of EPS from both strains was evaluated through albumin denaturation assay. HMX2-EPS and LP-Wang25-EPS exhibited albumin denaturation inhibition rates of 65.53 ± 2.57% and 71.32 ± 3.52%, respectively, showing a substantial anti-inflammatory activity. LP-Wang25-EPS simultaneously exhibited high anti-inflammatory activity and better anti-oxidative activity (DPPH scavenging activity: 93.75%; hydroxyl radical scavenging activity: 91.04%). EPS derived from other mutant strains exhibited antioxidant and anti-inflammatory activities distinct from those of the wild-type strains. The mutant *Cordyceps militaris* SU5-08 showed enhanced DPPH scavenging activity (Lin et al., 2012), and the mutant *Lactobacillus plantarum* GNS300 displayed enhanced antioxidant activity (Jae-Youn et al., 2023), whereas the mutant *Lactobacillus delbrueckii* subsp. *bulgaricus* EPSMLD exhibited reduced antioxidant activity (Adebayo-Tayo and Fashogbon, 2020). Oxidative stress is closely related to inflammatory responses, and reactive oxygen species can activate NF-κB and other inflammatory signaling pathways, promoting the release of pro-inflammatory cytokines (Morgan and Liu, 2011). Therefore, the strong anti-inflammatory activity of LP-Wang25-EPS may be associated with its high anti-oxidative activity, with these two properties acting synergistically to exert a cell-protective effect. Given that LP-Wang25 is a LAB with probiotic potential, this bioactive property may also be manifested in yogurt products. However, further studies are still required to confirm this beneficial effect on actual products.

### 3.4 Mutant strain LP-Wang25 enhanced yogurt quality

#### 3.4.1 *In situ* EPS production and rheological properties

To evaluate the application potential of yogurt fermentation, LP-Wang25 was fermented with a commercial starter culture (Yo-Mix® Prime 5.0) and *L. lactis* BL19 (Guo et al., 2026) with resulting yogurt samples designated as LPP5 and LPBL, respectively. Meanwhile, yogurt fermented solely by Prime-5.0 or *L. lactis* BL19 were used as controls, denoted as P5 and BL, respectively. In both fermentation systems (BL and P5), the introduction of the mutant strain LP-Wang25 significantly increased the EPS content of yogurt (*p* < 0.001) (Figure 3A). When *L. lactis* BL19 was used as the base starter culture, the EPS content increased from 71.99 to 135.41 mg/L, corresponding to an 88.1% increase. In contrast, in the commercial starter P5 system, the EPS content increased from 203.2 to 307.96 mg/L, representing a 51.6% increase.

**Figure 3:**
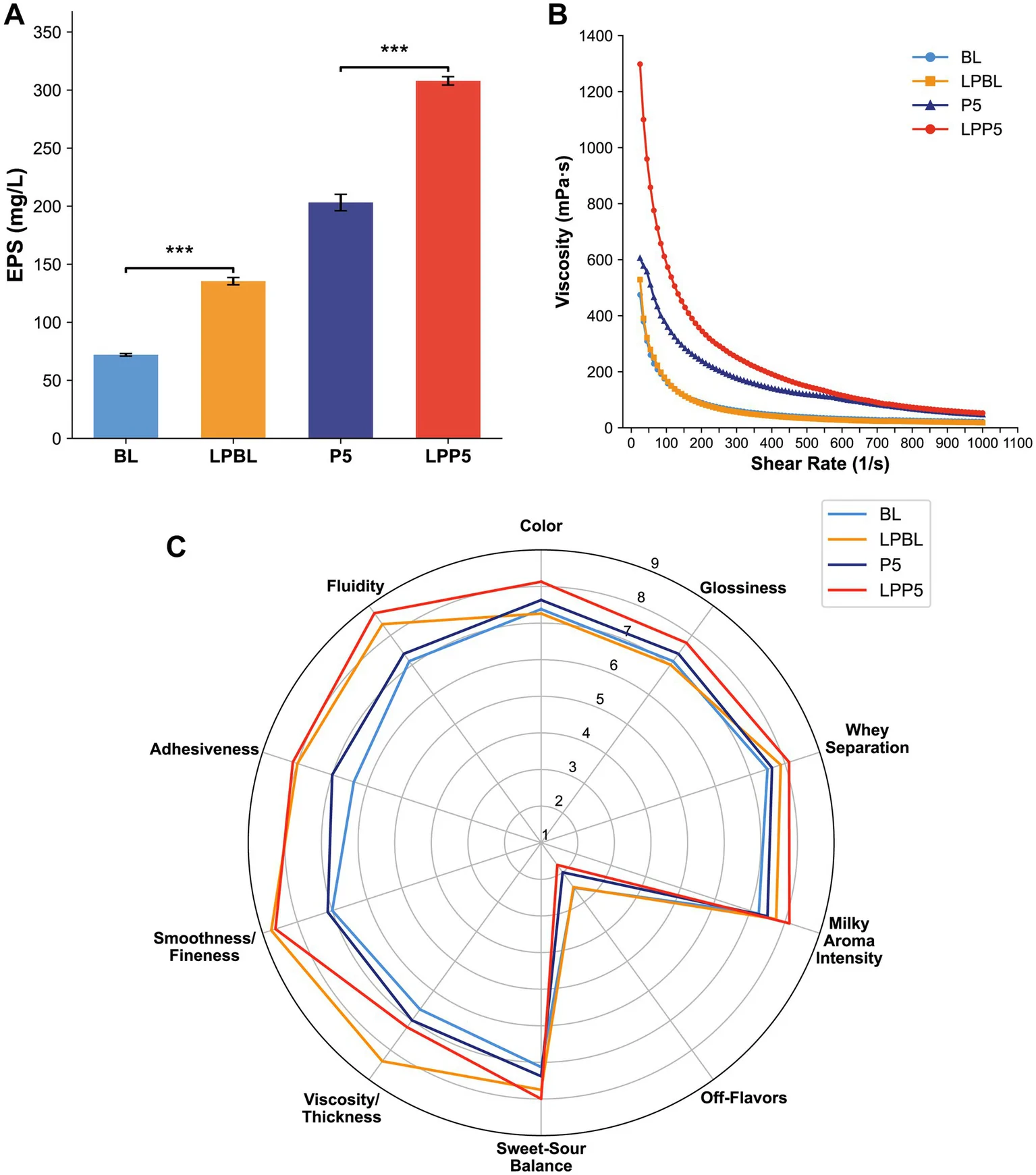
Comparison of **(A)** EPS production, **(B)** the apparent viscosity and **(C)** sensory evaluation scores of yogurts fermented by different combinations of starter cultures. BL: yogurt fermented by *L. lactis* BL19; LPBL: yogurt fermented by LP-Wang25 and *L. lactis* BL19; P5: yogurt fermented by Prime-5.0; LPP5: yogurt fermented by LP- Wang25 and Prime-5.0.

Meanwhile, rheological analysis showed that all groups exhibited typical shear dilution behavior, in which the apparent viscosity decreased with increasing shear rate (Figure 3B). This behavior was characteristic of pseudoplastic non-Newtonian fluids and was consistent with previously reported rheological models of yogurt systems (Zhao and Liang, 2022; Wang et al., 2025a). The apparent viscosity curves of BL and LPBL were nearly identical; however, the zero-shear viscosity of LPBL (529.21 mPa·s) was higher than that of BL (474.92 mPa·s). In contrast, the apparent viscosities of P5 and LPP5 were markedly higher than those of the other two yogurts, which may be associated with the increased EPS content in these samples (Figure 3A). Among the four groups, LPP5 exhibited the highest viscosity, with a zero-shear viscosity of 1298.15 mPa·s, corresponding to its highest EPS content. A similar relationship between EPS content and yogurt viscosity has been reported in previous studies. For example, Wang et al. (2025a) showed that yogurt fermented with the EPS-producing *L. plantarum* DPA1C reached its maximum EPS content on day 14 of storage, at which time the zero-shear viscosity also peaked. Likewise, Zhao and Liang (2022) found that higher EPS level resulted in increased viscosity. Wei et al. (2023) demonstrated that EPS could interact with casein, effectively filling the three-dimensional network of casein clusters and thereby enhancing yogurt viscosity. From a microstructural perspective, yogurts with higher EPS content exhibited fewer voids, more tightly associated casein micelles, and improved water-holding capacity and gel strength (Wang et al., 2023).

#### 3.4.2 Sensory evaluation

Sensory attributes play a critical role in consumer acceptance. In this study, quantitative descriptive analysis (QDA) was conducted on four yogurt samples, where the sensory scoring criteria are presented in Supplementary Table S5. Overall, the sensory score profiles indicated that the addition of the mutant strain improved key sensory attributes, specifically increasing viscosity and milky aroma while decreasing off-flavors (LPP5 vs. P5 and LPBL vs. BL) (Figure 3C). In terms of the total score, LPP5 achieved the highest score (74.62), followed by LPBL (73.66), both of which outperformed their corresponding base-fermented yogurts (Supplementary Table S6). For texture-related attributes, including viscosity, smoothness, and adhesiveness, the scores were enhanced with the addition of the mutant strain. Regarding fluidity, LPP5 and LPBL received higher scores, reflecting lower perceived flowability, which is consistent with their higher viscosity. A higher EPS content is generally associated with improved water-holding capacity and reduced whey separation. Accordingly, LPP5 exhibited the highest EPS content and therefore achieved the highest whey separation score, indicating the lowest degree whey separation. In terms of flavor attributes, the incorporation of the mutant strain increased the intensity of milky aroma, which may be associated with synergistic interactions among the mutant strain and the starter culture that enhance metabolic activity and alter the composition and concentration of flavor compounds. Wang et al. (2023) also reported that yogurt fermented by high EPS-producing strain has different volatile compounds compared with the control group. Nemati and Mozafarpour (2024) revealed that the addition of EPS increased the W5S, W1W and W2W value of e-nose, suggesting an increase in odor compounds. The increased milky aroma intensity attribute in the LPP5 sample was also consistent with the higher levels of 2,3-butandione and acetoin discussed in the flavoromics analysis below, both of which contribute characteristic milky and buttery notes. Besides, the denser network structure of the yogurt formed by increased EPS concentration can also influence the flavor profile. It has been reported that, in set yogurt supplemented with EPS, the contents of acids decreased, while other compounds, such as aldehydes and ketones, were present at relatively higher levels (Sun et al., 2026).

Overall, the sensory evaluation results demonstrate that the incorporation of the mutant strain LP-Wang25 significantly improved the sensory scores of the yogurt, particularly by enhancing viscosity, thereby contributing to a reduced reliance on pectin, gelatin and other hydrocolloids by *in situ* EPS production.

#### 3.4.3 Volatile flavor compounds analysis

The volatile flavor compounds in P5 and LPP5 were analyzed (Figure 4). LPP5 exhibited distinct red and blue spots in the spectral plot compared with the control sample (P5) (Figure 4A). Red spots represent volatile flavor compounds with higher concentrations than those in the control, whereas blue spots indicate lower concentrations. Compared with the control sample, the LPP5 sample showed increased diversity and abundance of volatile flavor compounds, suggesting that LP-Wang25 promoted the formation and accumulation of volatile flavor compounds.

**Figure 4:**
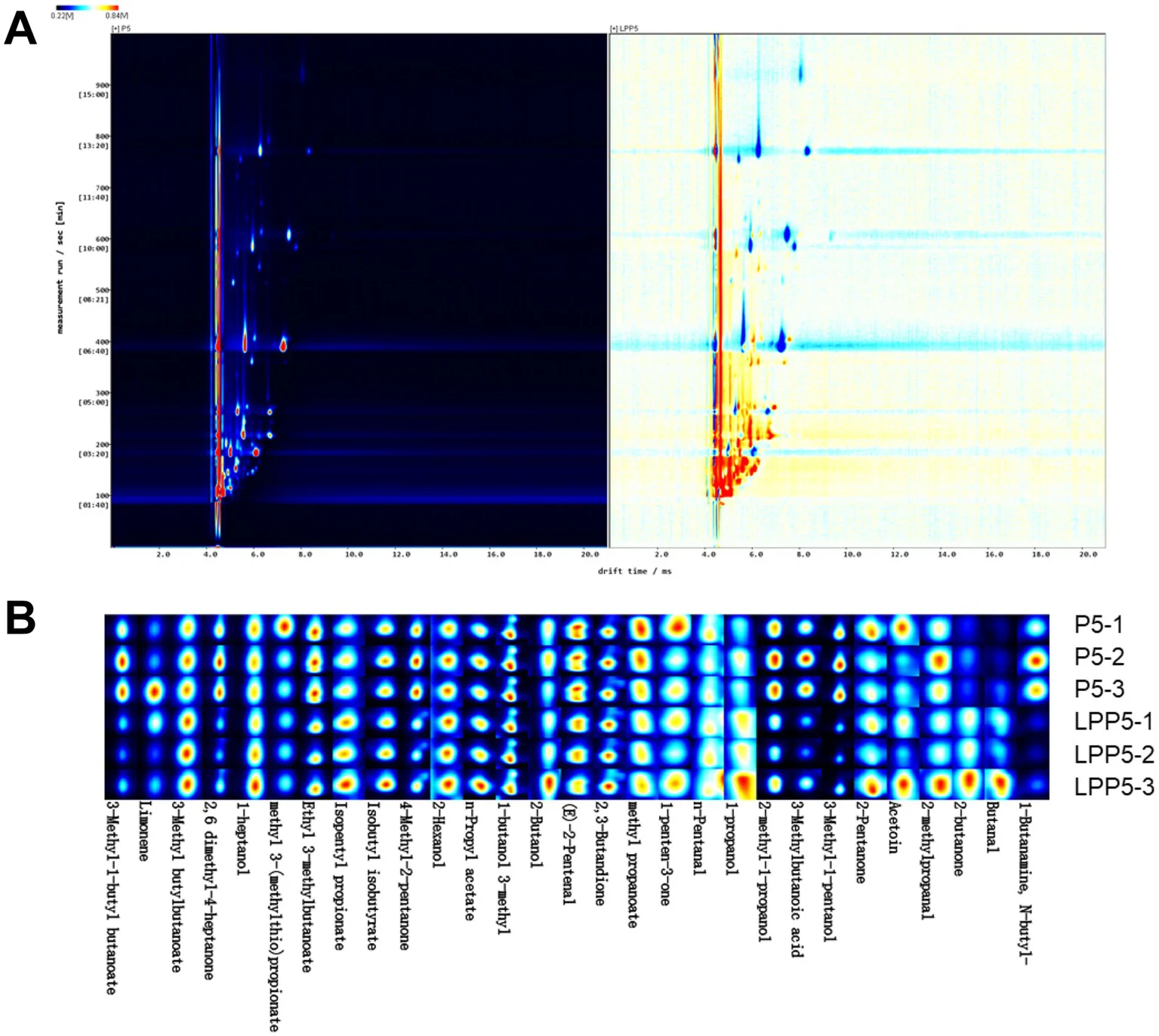
GC-IMS results for yogurt samples P5 and LPP5 **(A)** Difference plot with P5 spectra as reference (LPP5 vs. P5) **(B)** Ion mobility spectrum; brighter colors indicate higher concentrations.

More specifically, a total of 29 volatile flavor compounds were identified in P5 and LPP5 samples, comprising 3 aldehydes, 8 ketones, 7 alcohols, 1 acid, 6 esters, and 4 other compounds (Figure 4B). The concentrations of nine compounds were significantly lower in the LPP5 sample than in the P5 sample (*p* < 0.05) (Table 3). Among them, N-butyl-1-butanamine was associated with fishy and off-flavor notes, 3-methyl-1-pentanol contributed to cocoa- and malt-like aromas, high concentrations of 3-methylbutanoic acid were associated with rancid odors, and 2-methyl-1-propanol imparted malty and whisky-like notes. In contrast, the concentrations of four compounds were significantly increased in the LPP5 sample (*p* < 0.05) (Table 3). Among these compounds, 2-butanone contributed sweet and fruity notes, 1-propanol imparted fermented and slightly alcoholic aromas, and n-pentanal is associated with a fresh odor. The content of two key compounds, acetoin and 2,3-butanedione, were increased in the LPP5 samples. These two compounds are generally regarded as characteristic flavor compounds of yogurt and contribute pronounced milky and buttery notes (Lu et al., 2026; Tan et al., 2026b). These results suggest that fermentation involving LP-Wang25 promoted the formation of characteristic flavor compounds while suppressing the production of off-flavor compounds. Consequently, the LPP5 sample exhibited a fresher, milky, and fruity aroma profile. These results were consistent with the sensory evaluation, in which the LPP5 sample obtained higher score for milky aroma intensity and lower score of off-flavor.

**Table 3:** The peak areas (mean ± SD) of volatile flavor compounds identified by GC-IMS in P5 and LPP5 samples.

Compound	RI	CAS Number	Formula	Peak area	
				P5	LPP5
2,6-Dimethyl-4-heptanone	996.6	C108838	C9H18O	2561.25 ± 399.96*	1374.48 ± 229.36
Isobutyl isobutyrate	869.4	C97858	C8H16O2	1112.25 ± 69.45	1207.91 ± 144.35
4-Methyl-2-pentanone	784.5	C108101	C6H12O	3252.98 ± 302.58*	2447.13 ± 243.24
1-Butanol 3-methyl	746.4	C123513	C5H12O	6708.55 ± 499.70	6931.11 ± 444.28
1-Penten-3-one	714.7	C1629589	C5H8O	948.98 ± 231.95	939.77 ± 195.87
(E)-2-Pentenal	694.3	C1576870	C5H8O	4011.86 ± 50.67***	3021.38 ± 80.67
Methyl propanoate	600.7	C554121	C4H8O2	1290.08 ± 112.98	1152.76 ± 62.31
2-Butanol	606	C78922	C4H10O	623.82 ± 91.03	730.87 ± 188.49
1-Propanol	528	C71238	C3H8O	492.11 ± 63.61	745.29 ± 128.92*
2-Pentanone	733	C107879	C5H10O	1091.63 ± 136.18	1161.58 ± 278.06
2-Butanone	621	C78933	C4H8O	198.92 ± 30.63	485.30 ± 101.45**
2-Methylpropanal	589.1	C78842	C4H8O	258.78 ± 36.38	244.55 ± 72.63
3-Methyl-1-pentanol	889.4	C589355	C6H14O	10541.08 ± 1571.66**	4050.78 ± 1197.08
N-butyl-1-butanamine	994.6	C111922	C8H19N	447.78 ± 115.47*	131.81 ± 52.43
Acetoin	710.6	C513860	C4H8O2	509.53 ± 139.74	530.03 ± 225.10
2,3-Butandione	634	C431038	C4H6O2	3612.02 ± 309.45	4159.65 ± 377.03
Isopentyl propionate	901.7	C105680	C8H16O2	831.79 ± 47.25	1050.75 ± 115.31*
Methyl 3-(methylthio)propionate	962.1	C13532188	C5H10O2S	1010.69 ± 399.71	692.31 ± 12.95
Butanal	652.1	C123728	C4H8O	113.25 ± 48.81	393.69 ± 179.65
Limonene	1008.9	C138863	C10H16	1458.18 ± 961.62	1103.56 ± 435.01
3-Methyl butylbutanoate	1,012	C106274	C9H18O2	617.93 ± 44.07	680.13 ± 5.44
Ethyl 3-methylbutanoate	893	C108645	C7H14O2	8643.24 ± 437.73**	5387.60 ± 633.70
2-Hexanol	792.5	C626937	C6H14O	675.64 ± 43.44	756.05 ± 33.69
1-Heptanol	977.8	C111706	C7H16O	546.26 ± 13.75	560.39 ± 75.08
3-Methyl-1-butyl butanoate	1094.9	C106274	C9H18O2	2611.02 ± 563.70*	1153.59 ± 198.24
n-Propyl acetate	734.6	C109604	C5H10O2	2483.08 ± 300.06	2920.55 ± 478.15
n-Pentanal	709	C110623	C5H10O	861.77 ± 40.07	1023.64 ± 15.05**
3-Methylbutanoic acid	782.9	C503742	C5H10O2	2093.34 ± 462.93*	1076.00 ± 367.70
2-Methyl-1-propanol	686.7	C78831	C4H10O	8441.47 ± 995.69*	5211.22 ± 1129.70

Statistical significance was defined as: *p < 0.05, **p < 0.01, and ***p < 0.001.

Principal component analysis (PCA), with the first two principal components of the PCA accounting for 74.9% of the total variance, showed distinct distributions of P5 and LPP5 samples due to differences in the composition of volatile flavor compounds (Figure 5A). Correspondingly, the orthogonal partial least squares discriminant analysis (OPLS-DA) score plot further confirmed different volatile profiles of the two samples (Figure 5B). Eight key compounds discriminating the two yogurts were identified based on VIP > 1 and *p* < 0.05 (Supplementary Table S6). Among these compounds, 3-methyl-1-pentanol (wine, cocoa) (VIP = 2.43), 2-methyl-1-propanol (fresh, alcoholic, leather) (VIP = 2.14), and 3-methylbutanoic acid (sour, sweat, cheese) (VIP = 1.66) exhibited the highest discriminatory power. Notably, the concentrations of these three compounds were significantly decreased in the LPP5 sample (*p* < 0.05), indicating that the most pronounced differences between the two yogurts were associated with compounds contributing non-characteristic yogurt notes. The reduction therefore contributed to a purer and more typical yogurt flavor reflected in sensory evaluation.

**Figure 5:**
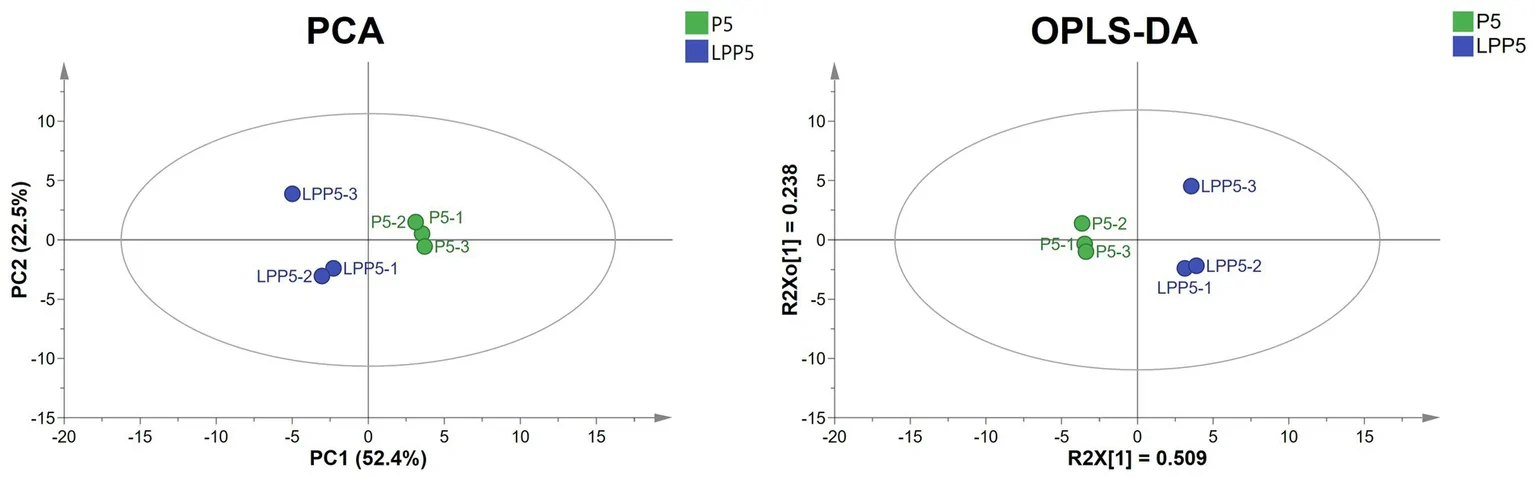
Principal component analysis (PCA) and orthogonal partial least squares discriminant analysis (OPLS-DA) of volatile compounds in P5 and LPP5 samples. **(A)** PCA score plot and **(B)** OPLS-DA score plot.

## 4 Conclusion

In this study, the growth performance, EPS production, genomic variations, functional bioactivities, and application potential in yogurt fermentation of a high-EPS-producing *L. plantarum* mutant strain LP-Wang25 were systematically investigated. The mutant strain demonstrated superior industrial traits, including a rapid growth rate, significantly enhanced EPS biosynthesis capabilities and notable antioxidant and anti-inflammatory activities. Genomic variation analysis revealed that space mutagenesis rendered a broad spectrum of mutations, primarily SNPs in non-coding regions. Interestingly, the gene clusters responsible for EPS synthesis via the Wzx/Wzy-dependent pathway remained conserved, suggesting that the EPS yield improvement likely resulted from transcriptional activity changes. Besides, the EPS produced by LPWang-25 exhibited higher DPPH and hydroxyl radical scavenging capacity, and better anti-inflammatory activity compared with the parental strain. Furthermore, yogurt fermentation showed that LP-Wang25 significantly increased the apparent viscosity, which consequently contributed to improved sensory attributes, including a smoother texture and a purer flavor, as further corroborated by GC-IMS analysis. Collectively, this work validates the potential of LP-Wang25 as a functional adjunct culture to enhance the overall quality of yogurts. Future research should focus on employing precision fermentation techniques, adaptive laboratory evolution for instance, to enhance and stabilize the quality-improving phenotypes of the mutant strain for more consistent industrial application.

## Data Availability

The raw sequencing data have been deposited in the NCBI BioProject database under accession number PRJNA1497584.
